# Achieving Lasting Umbilical Cord Decontamination and Sustainable Stem Cell Sourcing by Combining Plasma-Functionalized Liquid and Ultrasound

**DOI:** 10.3390/foods15030532

**Published:** 2026-02-03

**Authors:** Yuanyuan Pan, Alexander Pogoda, Monika Röntgen, Juergen F. Kolb, Sybille Hasse

**Affiliations:** 1 Department of Plasma Life Science, Leibniz Institute for Plasma Science and Technology e.V. (INP), Felix-Hausdorff-Str. 2, 17489 Greifswald, Germany; yuanyuan.pan@inp-greifswald.de (Y.P.); alexander.pogoda@inp-greifswald.de (A.P.); juergen.kolb@inp-greifswald.de (J.F.K.); 2 Work Group Cell Biology of Muscle Growth, Research Institute for Farm Animal Biology (FBN), Wilhelm-Stahl-Allee 2, 18196 Dummerstorf, Germany; roentgen@fbn-dummerstorf.de

**Keywords:** plasma-functionalized liquid, ultrasound, porcine umbilical cord, decontamination, cultured meat

## Abstract

The growing demand for alternative meat products is accelerating research into reproductive cell sources for cell-based meat processes, also called cultured meat. Porcine umbilical cord tissue is recognized as an advantageous source of mesenchymal stem cells (MSCs). However, effective decontamination must be achieved without compromising tissue integrity and cell recovery. In this study, we evaluated the decontamination of porcine umbilical cords using plasma-functionalized liquid (PFL) generated by a microwave-driven plasma source. It was applied alone and in combination with ultrasound, with the combined approach demonstrating superior performance. Specifically, the ultrasound–PFL combination treatment reduced the initial microbial load of individual tissue samples, ranging from 4.08 to 7.41 log_10_ CFU/g, approaching the limit of detection of the applied microbiological assays. Statistical analysis indicated a significant contribution of both PFL and ultrasound to microbial reduction, while mesenchymal stem cell yields (5.4 × 10^5^ cells/g tissue) and cell viability (84%) remained comparable to antibiotic-rinsed controls. Recovered cells retained functional capacity, as demonstrated by successful 3D spheroid formation. These results highlight ultrasound-assisted PFL rinsing as an efficient, long-lasting, and antibiotic-free decontamination strategy without compromising tissue compatibility. This study thereby extends the application of plasma-functionalized liquids and demonstrates the feasibility of sustainable stem cell sourcing. It offers opportunities in cultured meat bioprocessing.

## 1. Introduction

The increasing demand for sustainable and ethical alternatives to conventional meat production has driven significant advancements in cell-based meat, also known as cultured meat, describing meat that derives from cultured animal cells [1,2]. Among various cell sources that are explored for this application, mesenchymal stem cells (MSCs) derived from porcine umbilical cords (pUCs) have gained attention due to their non-invasive collection, high proliferation potential, and suitability for large-scale bioprocessing [3,4]. Unlike invasive muscle biopsies on live animals, pUCs represent a readily available alternative. However, one of the major challenges associated with pUC-based MSC isolation is microbial contamination, as pUCs are naturally exposed to environmental and commensal microorganisms during collection. High microbial loads can lead to contamination of cultures, affect cell viability and yield, and introduce variability in downstream applications for cultured meat production [5]. Ensuring effective and sustained decontamination without impairing the delicate pUC tissue is therefore essential for producing safe and high-quality stem cell preparations.

Antibiotics are commonly added to media or used during handling to reduce microbial contamination of biological tissues during stem cell isolation. However, increasing concerns about microbial resistance, regulatory restrictions, and environmental sustainability have prompted the search for alternative decontamination strategies [1,6]. Regulatory bodies, such as the European Food Safety Authority (EFSA) and the U.S. Food and Drug Administration (FDA), have imposed strict limitations on antibiotic use in food biotechnology, and it is conceivable that these limitations will equally apply to cultured meat in the future, thereby reinforcing the development of novel microbial inactivation methods. Several alternative decontamination strategies have been explored, including UV irradiation, organic acids, chlorine dioxide, bacteriophage therapy, and antimicrobial peptides [7,8,9,10]. However, many of these approaches are associated with drawbacks related to stem cell retrieval, such as cytotoxicity [11], limited antimicrobial effectiveness [12], or incompatibility with cell-based bioprocessing (heat inactivation), making them less suitable for the treatment of pUCs.

To address these challenges and limitations, plasma-functionalized liquids (PFLs) have recently emerged as a promising alternative to conventional decontamination methods, offering broad-spectrum antimicrobial activity with low cytotoxicity and good environmental compatibility [13,14,15,16]. These liquids are generated by exposing water or saline solutions to low-temperature plasma discharges. As a result, reactive oxygen and nitrogen species (RONS), including hydrogen peroxide (H_2_O_2_), ozone (O_3_), nitrite (NO_2_^−^), nitrate (NO_3_^−^), and hydroxyl radicals (•OH), accumulate in the liquid. Unlike antibiotics, PFLs exert their antimicrobial effects by inducing oxidative stress and by damaging multiple microbial targets simultaneously, reducing the likelihood of resistance development [17,18]. The decrease in pH and the presence of redox-active molecules in PFLs further enhance microbial inactivation while preserving biocompatibility with mammalian cells, making PFLs a promising alternative for pUC decontamination [13,19]. Although PFLs have already been successfully employed to improve food safety [20,21] and plant decontamination [22,23,24], their use in more complex applications, such as tissue processing, i.e., pUCs, remains underexplored. In our previous study, Pogoda et al. demonstrated that PFLs could effectively reduce microbial loads in pUC tissues [15]. The previously tested systems—two spark discharge devices and one flow dielectric barrier discharge (DBD)—differed in reactive species output, processing time, and scalability. Among them, the flow DBD showed the best microbial reduction (~3.55 log_10_ CFU/pUC). Despite these promising results, the approach had some limitations, such as the potential negative impact of PFL on MSC viability due to oxidative stress exposure mediated by RONS-containing solutions, resulting in reduced cell survival. To address the aforementioned limitations, the present study introduces an advanced decontamination strategy that combines PFL rinsing with ultrasound. This approach is designed to augment microbial inactivation and MSC recovery, as well as preservation. In order to achieve the objectives of this study, the investigation focused on two primary outcomes: sustained decontamination and the viability of the cells.

In continuation of our previous work and with large-scale application in mind, PFL was produced with a microwave-driven plasma source, which was originally developed to improve food hygiene [25,26]. Further improvement and potentially reduced oxidative stress damage on MSCs is expected from the combination of PFL with an ultrasound treatment, ultimately enhancing cell viability and recovery. Ultrasound has been widely recognized for its ability to improve bacterial removal through cavitation-induced microstreaming [27,28]. For food safety, ultrasound has been shown to enhance the efficiency of liquid-based treatments by disrupting microbial structures and increasing mass transfer effects [29]. Accordingly, we systematically compared four PFL-based decontamination strategies to optimize the treatment conditions for pUC-derived MSC isolation: (1) shaker-assisted PFL rinsing, (2) shaker-assisted PFL rinsing with recovery period, (3) ultrasound-assisted PFL rinsing alone, and (4) a combined ultrasound- and shaker-assisted PFL rinsing protocol.

The efficacy of these treatments was assessed by evaluating microbial inactivation efficiency, MSC yield, cell viability, and cellular functionality, as determined by a 3D spheroid formation assay. The combination of PFL rinsing and ultrasound treatment achieved MSC yields comparable to those obtained with conventional antibiotic protocols while also providing consistent and reproducible decontamination of pUCs. These findings represent a significant advancement toward the development of a scalable, antibiotic-free, and cell-friendly MSC processing method suitable for cultured meat production.

## 2. Materials and Methods

### 2.1. Sample Procurement and Preparation

Porcine umbilical cord samples were provided by the Research Institute for Farm Animal Biology (FBN, Dummerstorf, Germany) and transported to the Leibniz Institute for Plasma Science and Technology (INP) in 100 mL physiological sodium chloride solution (0.9% NaCl (*w*/*v*) with antibiotics (positive control, with 10% Penicillin/Streptomycin and 10% Amphotericin B; PAN-Biotech GmbH, Aidenbach, Germany) or without antibiotics (negative control and all samples for PFL rinsing). To minimize waste and in consideration of animal welfare, excessively long pUCs (approximately 50 cm) were randomly divided into two individual samples, which were treated as biological replicates and allocated to different rinsing strategies. In an effort to prevent cross-contamination, each sample was transported and rinsed in an individual sterile 250 mL Schott bottle with no shared containers or liquids.

### 2.2. Plasma Equipment Setup and Generation of Plasma-Functionalized Liquid (PFL)

PFL was generated by the microwave-driven plasma source (‘PLexc2’). Technical details are described elsewhere [30,31,32].

In brief, the microwave-driven discharge (frequency 2.54 GHz and power 1.3 kW) was operated with compressed air to generate plasma-processed air (PPA), which was then mixed with liquid in an international bulk container (IBC) of 1 m^3^ to produce PFL. For our application, 4 L of NaCl solution was converted into PFL in just 4 min, operating at a volume flow rate of 63 standard liters per minute (slm). During this process, the NaCl solution became enriched with long-lived reactive species, including hydrogen peroxide (H_2_O_2_), nitrate (NO_3_^−^), and nitrite (NO_2_^−^), and was immediately used in the rinsing protocols. Reactive species concentrations and pH values were monitored across batches (*n* = 3) and are summarized in Table 1.

### 2.3. Decontamination Treatments

In the current study, different rinsing protocols were developed, which involved PFL alone or PFL combined with ultrasound and shaking. Firstly, pre-rinsing was performed, which aimed at decontaminating the exterior surface of the entire pUC. Upon achievement of complete microbial decontamination in this step, an additional post-rinsing step was conducted before proceeding to the explant culture (see Section 2.5). Table 2 outlines the systematic experimental approaches conducted in this study, which included PFL rinsing with variation in exposure times, the addition of different recovery solutions, and the combination with shaker and ultrasound exposure. These variables were iteratively adjusted to identify optimal strategies that could effectively balance microbial decontamination and preservation of cell viability. An untreated NaCl solution (pH 6) served as a negative control, while a NaCl solution with antibiotics was included as a positive control. These controls served as benchmarks evaluating the effectiveness of the PFL treatments. Initially, 100 mL PFL alone was used as the rinsing solution at exposure times of 1, 5, and 10 min. This rinsing step was assisted by a shaker-incubator at 150 rpm (ES-80 Shaker-Incubator, Grant Bio, Cambridge, UK) to enhance the mechanical action. In the next iteration, pUCs were exposed to 100 mL NaCl solution or cell culture medium α-MEM with 10% FCS after PFL exposure to render cell integrity.

Finally, PFL rinsing was combined with ultrasound by placing pUCs in an ultrasonic bath (Sonorex Digiplus DL 255H, Bandelin Electronics GmbH & Co., Berlin, Germany; frequency 35 kHz; nominal power 160 W). The ultrasonic power density was adjusted to 53.3 W/L (hereafter referred to as 100%) or 26.7 W/L (hereafter referred to as 50%). The temperature in the ultrasonic bath was carefully maintained below 25 °C by adding ice as needed. Time intervals of 5 or 10 min were applied, and an intermittent shaking step was performed to increase the mechanical effect of the PFL on the pUCs. In addition, approximately 1 cm from both ends of the pUCs was trimmed off to minimize the risk of cross-contamination.

### 2.4. Microbial Assessment

Three independent cross-validation methods were used to confirm the absence of microbial residues or regrowth following PFL rinsing. First, pUC fragments were cut into smaller pieces using sterile scissors and forceps. Tissue (1.00 ± 0.01 g) was manually homogenized in 9 mL 0.1% peptone (peptone ex casein, Carl Roth GmbH & Co., Karlsruhe, Germany) for >30 s using a vortex mixer (Fisher Vortex Genie 2, Fisher Scientific Inc., New York, NY, USA) at high speed. The homogenate was serially diluted, and aliquots were plated on CASO agar using a spiral plater (Eddy Jet 2, IUL, Barcelona, Spain). For routine counts, 50 µL were plated (LOD = 200 CFU/g). When no colonies were observed at 50 µL, 1 mL of the undiluted homogenate was plated to improve sensitivity (methodological LOD = 1 CFU/mL), which, given 1 g in 10 mL, corresponds to 10 CFU/g. The plates were incubated at 37 °C for 24–48 h, and the colonies were counted and reported as log_10_ CFU/g.

Secondly, the remaining pUC samples were uniformly cut into tissue segments measuring 0.5 to 1 cm. The fragments were randomly placed with either side facing down onto CASO agar plates (Carl Roth GmbH & Co., Karlsruhe, Germany) and incubated at 37 °C for 48 h. A visual evaluation was performed. Three biological replicates and four technical replicates (two for the interior and two for the exterior surfaces) were performed.

Thirdly, CASO broth enrichment was performed to detect low levels of microbial contamination and slowly growing microorganisms with a detection limit of 1 CFU/pUC-fragments. pUC fragments (<0.5 × 0.5 cm tissue pieces) were transferred into 20 mL CASO broth (Carl Roth GmbH & Co., Karlsruhe, Germany) in 50 mL Falcon tubes and incubated at 37 °C. After 1 day (1 d) and after 7 days (7 d), a sample from the broth culture was subcultured on CASO agar plates, and colony-forming units (CFU/mL) were determined. Meanwhile, an internal quality control check for the PFL ensured that no additional contamination was introduced by the plasma system, with a detection limit of 1 CFU/mL.

### 2.5. Establishment of Explant Culture

Before the pUCs were processed for explant culture, a post-rinsing step was introduced to ensure complete decontamination of the internal surfaces of the pUCs and to raise the pH. For a single rinsing step, the pUCs were transferred to sterile 250 mL Schott bottles containing 10-fold diluted PFL without antibiotics. Rinsed pieces of pUC were placed in a cell culture dish (Ø 10 cm, Sarstedt AG & Co., Nümbrecht, Germany) with the inner side facing downwards. Excess water was aspirated prior to carefully adding 10 mL of explant medium (αMEM with 10% FCS with or without 1% Penicillin/Streptomycin and 1% Amphotericin B (all PAN-Biotech GmbH, Aidenbach, Germany)). The explants were incubated at 37 °C with 5% CO_2_. On day 8, the tissue pieces were removed, and 10 mL of pre-warmed culture medium was added to continue the culture. The culture medium was replaced twice a week, with the final culture period extending to 19 days. Images of the pUC-derived MSCs were captured for morphological characterization using a Zeiss Axiovert 40 CFL microscope (Carl Zeiss, Jena, Germany) with a 10× magnification objective coupled with a high-resolution Axiocam-40 CFL digital camera (Carl Zeiss, Jena, Germany).

On day 19, the culture medium was aspirated, and the cells were washed twice with 1× DPBS (without calcium and magnesium, PAN-Biotech GmbH, Aidenbach, Germany). The cells were detached by incubation with 5 mL of Accutase^®^ enzyme (Accutase Cell Detachment Solution, Biolegend, San Diego, CA, USA). After detachment, an additional 1 mL of DPBS was used to rinse the cell plate again. The cell suspension was then centrifuged at 500 rpm for 8 min. The supernatant was carefully removed, and the cells were resuspended in 1 mL of explant medium. For both the antibiotic-rinsed and PFL-rinsed cell groups, 20 µL of the resuspended cells was mixed with 180 µL of 4,6-diamidino-2-phenylindole (DAPI, 1 µM, Sigma-Aldrich, Taufkirchen, Germany) to determine cell viability. Using a CytoFLEX-S flow cytometer (Beckman Coulter, Brea, CA, USA), the number of live (unstained) and dead (DAPI-positive) cells was immediately counted. Meanwhile, an additional 20 µL of the antibiotic-rinsed cell suspension was mixed with 180 µL DPBS, serving as an internal reference. A total of 100,000 gated events were captured and displayed for each sample.

### 2.6. Establishment of 3D Cell Culture

On day 19, MSCs derived from pUCs (passage 0) were harvested as described above. Then, 2 × 10^3^ cells were seeded into each well of a Nunclon Sphera 96-well U-bottom plate (Thermo Fisher Scientific, Roskilde, Denmark) and overlaid with 100 µL culture medium. The well plate was centrifuged at 800 rpm for 5 min to promote cellular aggregation, then incubated at 37 °C with 5% CO_2_ for 3 and 5 days. Half of the plating medium was replaced on day 3. The morphology of the spheroids was captured using a Zeiss Axiovert 40 CFL microscope (Carl Zeiss, Jena, Germany) equipped with a 5× magnification objective and a high-resolution Axiocam-40 CFL digital camera (model Axiocam MRC; Carl Zeiss, Jena, Germany). ImageJ 1.54g (National Institutes of Health (NIH), Bethesda, ML, USA) was applied to assess the Feret diameter in µm.

### 2.7. Data Analysis

All statistical analyses were performed in OriginPro^®^ 2022b (OriginLab Inc., Northampton, MA, USA). For CFU outcomes, groups that reached the assay’s lower limit of detection (LOD) (no colonies after enrichment) were evaluated on the binary endpoint (growth vs. no growth) using Fisher’s exact test (two-sided), with Clopper–Pearson 95% confidence intervals (CIs) for sterility proportions; pairwise comparisons were adjusted for multiple testing using Holm’s step-down procedure to control the family-wise error rate (FWER). LODs were 10 CFU/g for CASO agar plating and 1 CFU per pUC fragment for CASO broth enrichment (sterility defined as <LOD). For broth enrichment panels, the LOD coincides with the axis origin (log_10_ = 0) and was therefore not shown. For non-sterile CFU distributions, data were analyzed non-parametrically using Kruskal–Wallis with Dunn’s multiple-comparison procedure. Continuous outcomes (cell yield, cell viability) were compared by one-way ANOVA with Fisher’s least significant difference (LSD) post hoc test at α = 0.05. Figures are shown as boxplots with median and interquartile range (IQR; 25th–75th percentile). Whiskers follow Tukey’s rule (with 1.5 × IQR of Q1 and Q3), and points beyond the whiskers are plotted as outliers. All individual data points are overlaid as jittered dots. Mean values are displayed only for continuous outcomes (cell yield and viability).

## 3. Results

### 3.1. Decontamination Efficacy Increases with Rinsing Time

**Figure 1:**
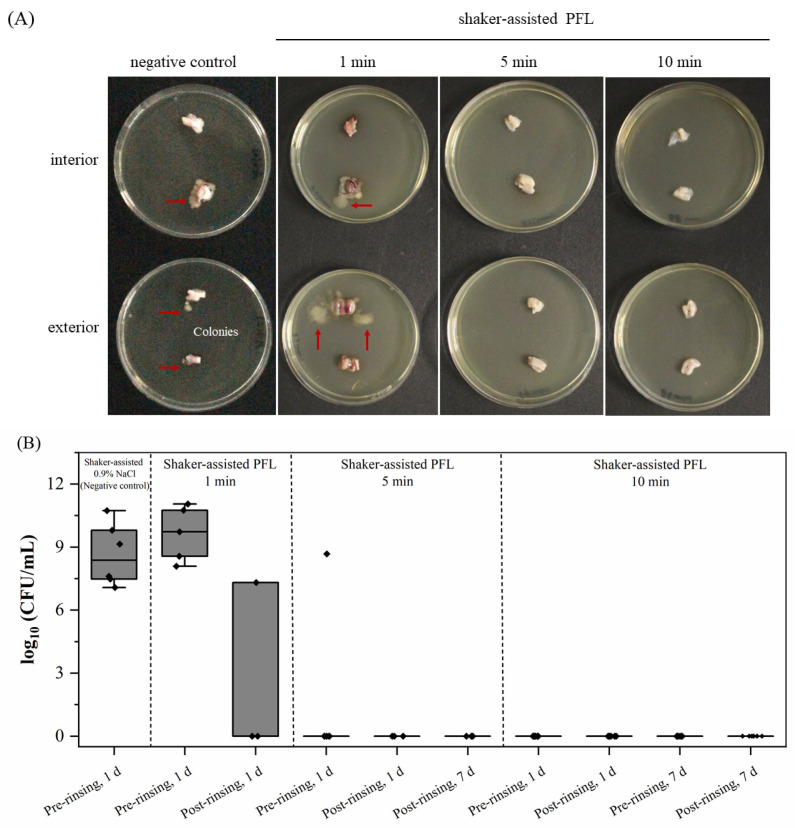
Representative tissue samples incubated on CASO agar plates after shaker-assisted PFL rinsing for 1, 5, and 10 min or rinsing with untreated 0.9% NaCl solution (negative control) (**A**). Red arrows indicate visible bacterial colonies on both the interior and exterior surfaces of the pUC segments for the negative control and for 1 min rinsing in PFL. In contrast, no bacterial growth is visible for samples after shaker-assisted PFL rinsing for 5 min and 10 min. (**B**) Microbial load (log_10_ CFU/mL) obtained from cultivation of pUC samples in CASO broth after rinsing. Rinsing with PFL for 5 and 10 min achieved a significant reduction in microbial load as opposed to rinsing for 1 min and the negative control. The reduction in microbial load was sustained even after prolonged incubation time (post-rinsing 1 d and 7 d). Boxplots show median, interquartile range (IQR; 25th–75th percentile), and whiskers (with 1.5 × IQR of Q1 and Q3), with all individual values overlaid as jittered dots. Post-rinsing (1 d) sterility: NaCl 0/6, PFL 1 min 2/3, PFL 5 min 3/3, and PFL 10 min 6/6; omnibus 2 × 4 χ^2^ = 15.19; *p* = 0.00166. Pairwise Fisher (two-sided) vs. NaCl: 1 min *p* = 0.0833; 5 min *p* = 0.0119; and 10 min *p* = 0.00217. Sterility (95% Clopper–Pearson CIs): NaCl (post 1 d): 0/6 (95% CI 0.0–45.9%); PFL 1 min (post 1 d): 2/3 (95% CI 9.4–99.2%); PFL 5 min (post 1 d): 3/3 (95% CI 29.2–100.0%); PFL 10 min (post 1 d): 6/6 (95% CI 54.1–100.0%). Pairwise Fisher (two-sided) vs. NaCl (Holm-adjusted): 1 min *p* = 0.0833; 5 min *p* = 0.0238; 10 min *p* = 0.0065.

After rinsing, pUC segments were incubated on agar plates, and bacterial colonies were detected in samples of the negative controls, exposed to NaCl solution (Figure 1A). When a 1 min PFL rinse was applied, bacterial colonies were also observed on 8 out of 12 pUC segments. In contrast, no bacterial growth was detected on the agar plates containing segments rinsed with PFL for 5 min or 10 min, demonstrating the antimicrobial efficacy for the extended rinsing times.

To cross-validate these findings, CASO broth enrichment for up to 7 days was utilized to identify any remaining viable microorganisms that might have survived the PFL treatment (Figure 1B). Consistent with the aforementioned findings, all samples from the negative control (NaCl rinsing) showed significant microbial growth (5.89 × 10^9^ CFU/mL (±8.92)) after incubation at 37 °C for one day. However, for the 5 min PFL rinse, 5 out of 6 samples showed no bacterial colonies after incubation, and no microbial growth was observed after post-rinsing, even after extending the incubation period to 7 days, indicating effective decontamination. The 10 min PFL rinse further confirmed the decontamination efficacy, with no culturable bacteria detected in any of the samples after the pre- or post-rinsing steps, even following prolonged incubation. In contrast, for the 1 min PFL rinse, microbial growth was detected in all samples in CASO broth after one day, and even after post-rinsing, 33% of the samples still showed bacterial presence (Figure 1B). This confirms that a 1 min PFL rinse is insufficient for complete decontamination, but the 5 min and 10 min PFL rinsing protocols effectively achieved aseptic conditions on pUCs when performed in multiple rounds, as validated across various experimental batches. However, despite achieving effective microbial decontamination, the procedure proved highly detrimental to cell viability. Consequently, no adherent cells were recovered, even when the culture duration was extended to 18 days. Hence, this finding prompted us to implement additional measures and further refine the protocol in order to achieve the objectives of this study, including sustained tissue decontamination and sufficient viability of isolated cells.

### 3.2. Introducing a Recovery Period Restores Cell Viability

**Figure 2:**
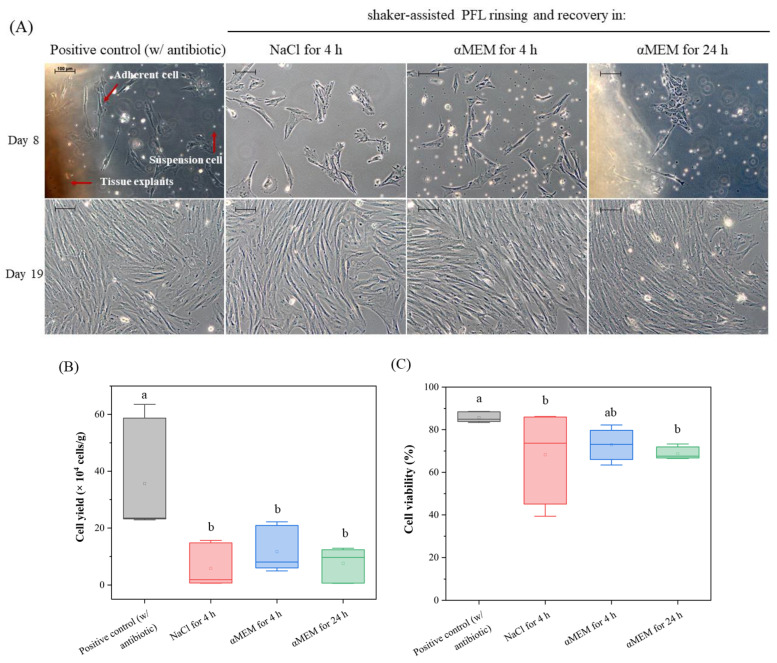
Explant culture of pUCs after implementing different recovery solutions. (**A**) Representative images of cell cultures after shaker-assisted PFL rinsing for 10 min, followed by immersion in recovery solutions on day 8 (top row). The bottom row displays the same culture after 19 days of incubation. Uniformly spread cells at high density were observed for all treatment conditions. Scale bar equals 100 µm. (**B**) Cell yields for different recovery scenarios. The positive control (with antibiotics) showed a significantly higher cell yield compared to the PFL-treated samples in recovery solutions. (**C**) Cell viability after PFL rinsing and incubation in recovery solutions. Boxplots show mean (empty square), median (line), and interquartile range (IQR; 25th–75th percentile) with whiskers (with 1.5 × IQR of Q1 and Q3) (*n* = 4). Statistics: one-way ANOVA + Fisher’s LSD (α = 0.05); significant differences are indicated by different letters (a, b) above the bars.

To overcome the harsh impact and the potentially damaging effects of PFL on tissue integrity, we evaluated the effectiveness of different recovery solutions applied for 4 h and 24 h (Figure 2A). The pUC samples were pre-rinsed with PFL for three cycles of 10 min each, as outlined in Table 2. Samples of pUC were subsequently transferred to either a NaCl solution or to a nutrient-rich cell culture medium (αMEM supplemented with 10% FCS) for 4 h or 24 h before proceeding with post-rinsing. Only post-rinsed pUC pieces that achieved sterility were processed to create explant cultures. As shown in Figure 2A, tissue explant cultures exhibited a mix of fibroblast-like cells, irregularly shaped cells, and suspension cells in all groups of the recovery solution and the positive control on day 8. The adherent cells began growing out from the tissue explants in a dispersed pattern. Cell density increased over time, and cells grew to confluency by day 19.

The final cell yield across the different recovery solutions varied notably, as presented in Figure 2B, with the highest yields for the positive control (35.6 × 10^4^ cells/g (±19.2)) and the lowest for the NaCl solution after 4 h (5.88 × 10^4^ cells/g (±7.13)). Recovery in αMEM with 10% FCS for 4 h and 24 h yielded 11.7 × 10^4^ cells/g (±7.6) and 7.62 × 10^4^ cells/g (±5.55), respectively. Statistical analysis using one-way ANOVA revealed a significant difference between the positive control and all groups from the recovery solution in terms of cell yield (*p* < 0.05). No significant differences were detected among the different recovery solutions (*p* > 0.05).

The cell viability for the recovery solutions is summarized in Figure 2C. The mean cell viability values for the positive control, NaCl solution for 4 h, and αMEM with 10% FCS for 4 h and for 24 h were 85.69% (±0.93), 68.23% (±11.10), 72.94% (±2.91), and 68.74% (±1.54), respectively. Statistical analysis indicated a significant decrease in cell viability for immersion in NaCl solution for 4 h and αMEM with 10% FCS for 24 h compared to the positive control (*p* < 0.05). However, no significant difference was observed for immersion in αMEM with 10% FCS for 4 h compared to the positive control (*p* > 0.05), suggesting that the recovery method involving a 4 h recovery period in αMEM with 10% FCS and reduced post-rinsing steps was more effective in maintaining cell viability.

These findings confirmed that PFL rinsing might negatively affect cell viability. A 4 h recovery period in αMEM with 10% FCS appeared to be the most effective procedure in mitigating these effects and demonstrated the best preservation of cell viability among the PFL-treated samples. Nevertheless, even with improved cell viability, the cell yield in the PFL-treated scenarios was diminished compared to that in the positive control, emphasizing the need for further optimization of the PFL rinsing process to improve outcomes.

### 3.3. Ultrasound-Assisted PFL Rinsing Eliminates Bacterial Contamination but Yields Low Cell Numbers

Further optimization of the protocol involved ultrasound-assisted rinsing steps during the process to maintain antimicrobial effectivity and to simultaneously improve the cell yield, as outlined in Table 2.

**Figure 3:**
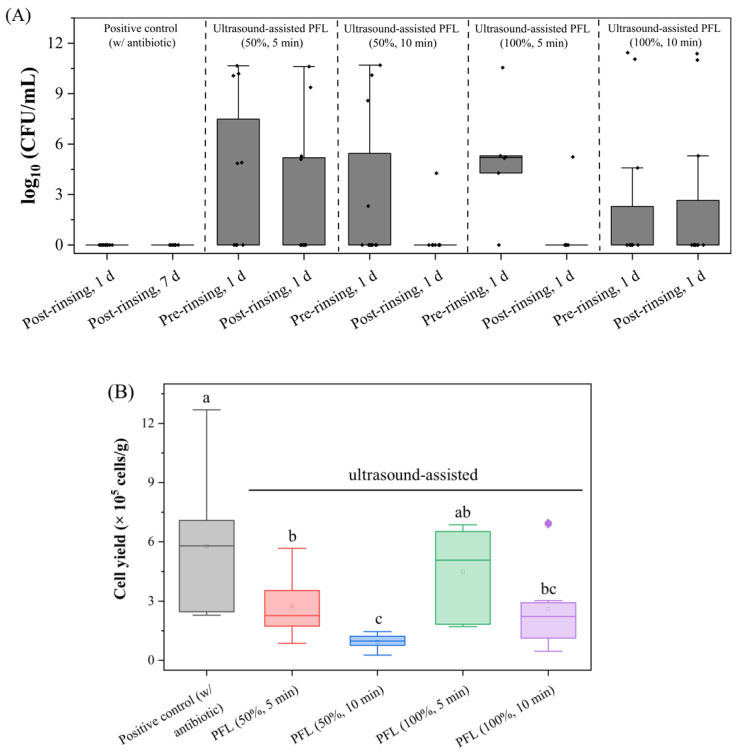
Antimicrobial efficacy and cell yield after ultrasound-assisted PFL rinsing. (**A**) Microbial load (log_10_ CFU/mL) after rinsing for 5 and 10 min, with PFL in combination with ultrasound treatment operated at 50% and 100% intensity. Boxplots show the microbial load after pre-rinsing in PFL with ultrasound and after post-rinsing treatments for an incubation of 1 day in CASO broth (post-rinsing, 1 d) and, in the case of the positive control, also for 7 days (post-rinsing, 7d). Boxplots show median and interquartile range (IQR; 25th–75th percentile) with whiskers (with 1.5 × IQR of Q1 and Q3). Sterility counts post-rinsing (1 d): antibiotic 12/12, US-PFL 50% 5 min 8/12, US-PFL 50% 10 min 11/12, US-PFL 100% 5 min 5/6, and US-PFL 100% 10 min 9/12; omnibus 2 × 6 χ^2^ = 6.00, *p* = 0.306. Pairwise Fisher (two-sided) vs. antibiotic: *p* = 0.333, 0.093, 1.000, 0.333, and 0.217, respectively. Sterility (95% Clopper–Pearson CIs): antibiotic 12/12 (95% CI 73.5–100.0%); US-PFL 5 min 50% 8/12 (95% CI 38.5–90.6%); US-PFL 10 min 50% 11/12 (95% CI 62.7–99.7%); US-PFL 5 min 100% 5/6 (95% CI 39.8–99.4%); US-PFL 10 min 100% 9/12 (95% CI 47.9–95.4%); Pairwise Fisher (two-sided) vs. antibiotic (Holm-adjusted): 50%–5 min *p* = 0.373, 50%–10 min *p* = 1.000, 100%–5 min *p* = 0.667, and 100%–10 min *p* = 0.652. (**B**) Comparison of cell yield (×10^5^ cells/g) for the positive control and the different ultrasound-assisted PFL-treated strategies. Boxplots show mean (empty square), median (line), and interquartile range (IQR; 25th–75th percentile) with whiskers (with 1.5 × IQR of Q1 and Q3) (*n* = 4). Statistics: one-way ANOVA + Fisher’s LSD (α = 0.05); significant differences are indicated by different letters (a, b, c) above the bars.

The microbial load (log_10_ CFU/mL) was assessed to evaluate the decontamination efficiency of PFL treatments applied with different ultrasound power settings, namely, 50% ultrasound power for 5 and 10 min and 100% ultrasound power for 5 and 10 min, in comparison to the positive control using antibiotics. The findings are summarized in Figure 3A. The positive control showed complete microbial reduction, with no detectable colonies after rinsing. Treatment with 50% ultrasound power for 5 min showed no substantial reduction in microbial load, indicating that these conditions were not sufficient to fully eliminate microbial contamination (Figure 3A). An extended exposure of 10 min did not result in a considerable improvement. In contrast, the application of 100% ultrasound power for 5 min achieved a strong reduction in microbial load, almost approaching the sterility levels of the positive control. With 100% ultrasound power for 10 min, individual samples showed microbial counts; therefore, this procedure did not achieve complete sterility in every sample. These findings indicate that higher ultrasound power is crucial for enhancing the effectiveness of PFL for microbial reduction, regardless of the treatment duration.

In Figure 3B, the cell yield (cells/g pUC) was analyzed for different treatment strategies to determine the impact of ultrasound-assisted PFL rinsing on cell recovery. The highest cell yield was detected for the positive control, reaching 5.78 × 10^5^ cells/g (±2.95). This was significantly higher than for all pUCs after ultrasound-assisted PFL rinsing, as indicated by statistical analysis. The cell yield for pUCs rinsed with ultrasound-assisted PFL for 5 and 10 min reached 2.74 × 10^5^ cells/g (±1.56) and 0.73 × 10^5^ cells/g (±3.66), respectively, for 50% ultrasound power, and 4.50 × 10^5^ cells/g (±2.22) and 2.61 × 10^5^ cells/g (±2.18), respectively, for 100% ultrasound power.

### 3.4. Combination of Ultrasound- and Shaker-Assisted Rinsing Optimized Decontamination Efficiency and Cell Yield

In preliminary experiments, contamination levels in different sections of the pUC were analyzed, which revealed that the contamination at both ends of the pUC was 5–10 times higher than in the middle section. To minimize the risk of cross-contamination during handling, approximately 1 cm was removed from each end of the pUC before the rinsing processes began. This precautionary step effectively reduced the spread of contaminants, thereby enhancing the decontamination processes’ overall efficiency. To achieve 100% decontamination efficiency, a combined approach using alternating ultrasound- and shaker-assisted PFL rinsing was explored.

**Figure 4:**
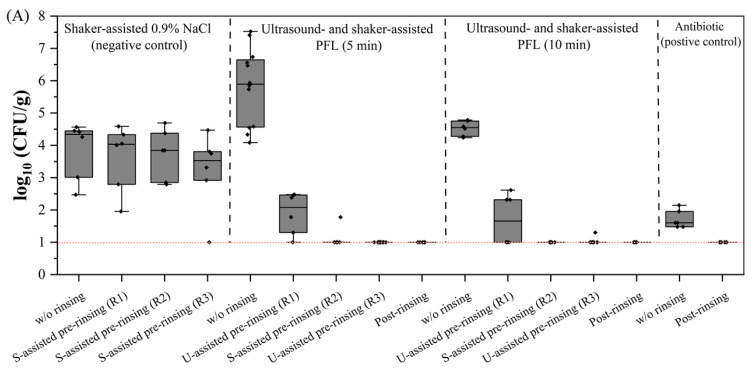

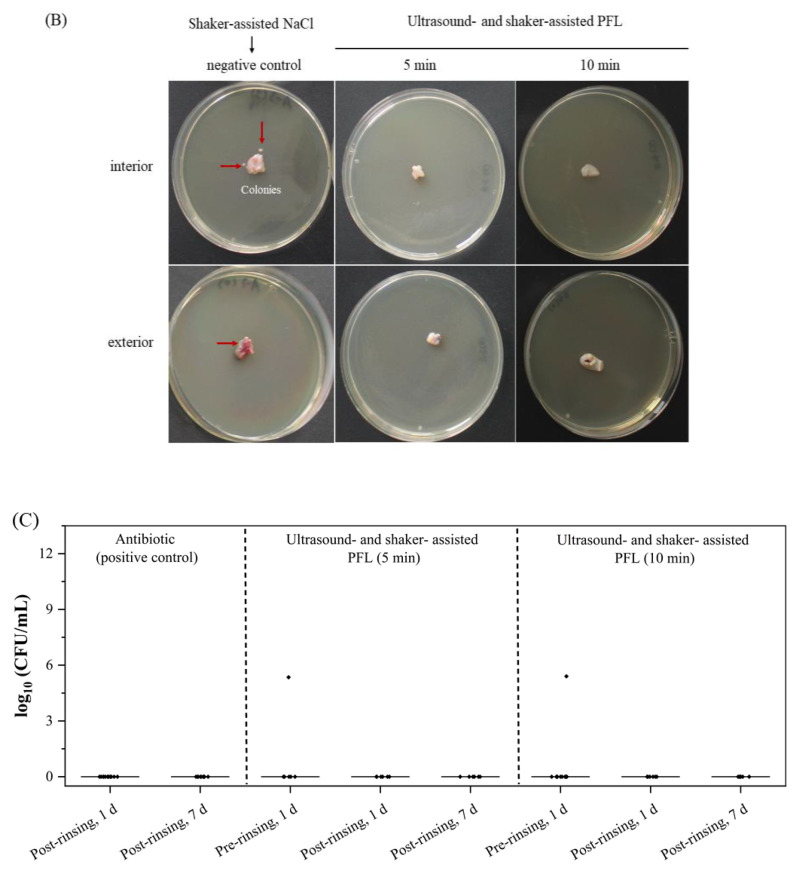
Microbial load (log_10_ CFU/g) for a combined ultrasound-assisted and shaker-assisted PFL treatment. (**A**) The data show a significant reduction in microbial load following the PFL treatments with alternating ultrasound and shaking for 5 min and 10 min compared to the negative control. The boxplots indicate the microbial load before and after each round of rinsing (R1 to R3), and after post-rinsing. (R1: round 1; R2: round 2; R3: round 3; S: shaker; U: ultrasound). No microbial growth in post-rinsed samples indicated a lasting effect. Dotted line indicates LOD (10 CFU/g; log_10_ = 1.00). Boxplots show the interquartile range (IQR; 25th–75th percentile) with the median line; whiskers follow the Tukey rule (1.5 × IQR of Q1 and Q3), and outliers beyond whiskers are plotted; all individual values are overlaid as jittered points. A horizontal dotted line represents LOD for agar plating (10 CFU/g; log_10_ = 1.00). Pre-rinse distributions: 5 min Kruskal–Wallis H = 6.72, *p* = 0.035; 10 min H = 3.23, *p* = 0.199. Post-rinsing sterility: 5 min 6/6, 10 min 6/6, antibiotic 12/12; omnibus 2 × 3 χ^2^ = 0.140, *p* = 0.933; pairwise Fisher vs. antibiotic *p* = 1.000 for both comparisons. (**B**) Decontamination effectiveness for interior and exterior sections of the pUCs samples after incubation on CASO agar plates. The presence of microbial colonies was visible in the negative control rinsed with untreated NaCl solution on both the interior and exterior surfaces, while the alternating ultrasound-assisted and shaker-assisted PFL-treated samples showed no visible bacterial growth. Colonies are indicated by red arrows. (**C**) Lasting antimicrobial effects were obtained by incubation of rinsed pUC samples (R1 to R3) in CASO broth for 1 or 7 days (log_10_ CFU/mL). While the occasional CFU was detected after pre-rinsing with ultrasound- and shaker-assisted rinsing steps, the final rinsing step (post-rinsing) completely eliminated bacterial growth even for incubation of 7 days (7 d). No CFUs were detected for the antibiotic-treated positive control. Post-rinsing sterility at 1 d: antibiotic 12/12, US-PFL 5 min 6/6, and US-PFL 10 min 6/6; at 7 d: antibiotic 12/12, US-PFL 5 min 6/6, and US-PFL 10 min 6/6. Pairwise Fisher’s exact tests (two-sided) vs. antibiotic: *p* = 1.000 for both comparisons.

The combined approach involved three rinsing steps in PFL: starting with an ultrasound step (R1), followed by a shaker-assisted rinse (R2), and finishing with another ultrasound step (R3). Upon achieving complete decontamination, post-rinsing procedures began. Figure 4A shows the bacterial load (log_10_ CFU/g) of minced pUC samples under the tested rinsing strategies. Rinsing three times with NaCl solution alone (negative control) consistently resulted in high bacterial loads with log_10_ 3.86 to 3.04 CFU/g. In contrast, alternating ultrasound- and shaker-assisted steps for 5 min each led to a significant bacterial load reduction—from 5.81 down to 1.90 log_10_ CFU/g—only after the first rinse (R1), which showed a lasting effect resulting in undetectable bacterial growth in subsequent rounds (R3 to post-rinsing). Similar results were achieved for alternating ultrasound- and shaker-assisted steps for 10 min, each leading to a significant bacterial load reduction—from 4.52 to 1.21 log_10_ CFU/g—after the first rinse (R1), with persistent effectiveness. As expected, the positive control was free of bacteria after post-rinsing.

Figure 4B illustrates the results of pre-rinsed pUC fragments incubated on agar plates after the combined rinsing procedure. The red arrows indicate bacterial colonies, which were only abundant in the negative control. Conversely, the absence of colonies in samples from alternating ultrasound- and shaker-assisted PFL rinsing indicated successful elimination of bacterial contamination on interior and exterior surfaces of pUC fragments.

A further incubation of tissue samples in CASO broth, used to culture residual bacteria, confirmed complete decontamination, as illustrated in Figure 4C. Occasionally, bacterial growth was detected after pre-rinsing. However, in post-rinsed samples, the bacterial load (log_10_ CFU/mL) was entirely eliminated when alternating ultrasound- and shaker-assisted steps were applied for 5 min and 10 min, reaching the levels of antibiotic-treated samples.

### 3.5. Successful Cell Isolation and Spheroid Formation Confirms MSC Functionality

**Figure 5:**
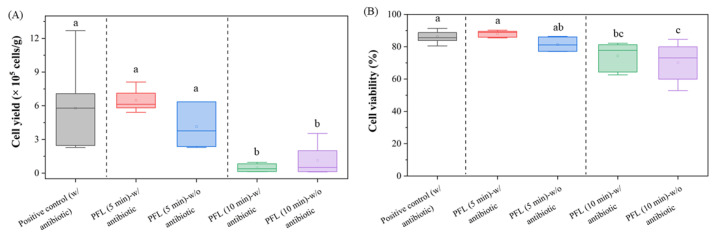
Comparison of cell yield and cell viability for combined ultrasound- and shaker-assisted rinsing steps. (**A**) Cell yield (×10^5^ cells/g) for the positive control and PFL treatments in combination with ultrasound and shaking for 5 min and 10 min. Significant differences are indicated by different letters (a, b) at α = 0.05. (**B**) Cell viability (%) after the combined rinsing steps. Statistical differences between groups are indicated by different letters (a, ab, bc, c). Note: Cell yield and cell viability for alternating ultrasound- and shaker-assisted PFL rinsing for 5 min were as high as for the positive control. Boxplots show the interquartile range (IQR; 25th–75th percentile) with the median line and mean (empty square); whiskers (1.5 × IQR of Q1 and Q3).

The combined ultrasound- and shaker-assisted PFL rinsing revealed successful isolation of viable cells during explant culture. As shown in Figure 5A, cells were harvested from all tested samples, although the cell yield varied between different conditions. The final cell yields for the positive control and alternating ultrasound- and shaker-assisted PFL rinsing for 5 min were 5.78 × 10^5^ cells/g (±2.95) and 5.45 × 10^5^ cells/g (±1.94), respectively. Alternating ultrasound- and shaker-assisted PFL rinsing for 10 min revealed a lower cell yield of 1.14 × 10^5^ cells/g (±1.33). In parallel, the cell culture was performed with the addition of antibiotics to the culture medium, which produced cell yields of 6.24 × 10^5^ cells/g (±0.71) and 0.47 × 10^5^ cells/g (±3.37) for the 5 min and 10 min treatments, respectively.

Figure 5B presents the cell viability percentage for alternating ultrasound- and shaker-assisted PFL rinsing for 5 and 10 min, with and without antibiotics in the culture medium. The positive control and shorter PFL rinses (5 min) resulted in higher cell viabilities of 86.23% (±0.68), 89.26% (±0.35), and 84.24% (±1.21) compared to 10 min rinses (74.36% (±3.44) and 70.15% (±3.66)).

As anticipated, the use of antibiotics in all rinsing steps resulted in high final cell yield and good cell viability (positive control). The antibiotic-free group revealed cell yields and cell viabilities as high as the positive control (*p* > 0.05), indicating good preservation of cell viability after 5 min of alternating ultrasound- and shaker-assisted PFL rinsing. However, a significant reduction in cell yield and viability was noted after the same treatment regimen for 10 min compared to the positive control (*p* < 0.05) (Figure 5B).

**Figure 6:**
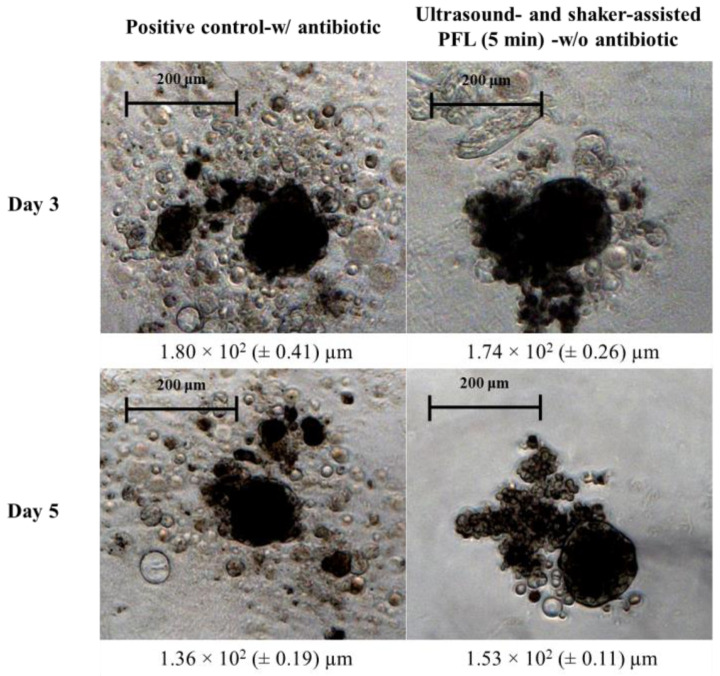
Spheroid culture (3D) derived from mesenchymal stem cells (MSCs) for day 3 (top row) and day 5 (bottom row). In the left column, the cells derived from the positive control (with antibiotics) and the spheroids appear dense and compact, demonstrating strong cell–cell adhesion. The right column shows the formation of spheroids of cells after ultrasound- and shaker-assisted PFL treatment protocol (for a treatment of 5 min). The spheroids demonstrated comparable diameters to the positive control. The Feret diameter is indicated below each image (*n* = 5).

To evaluate the functionality of isolated MSCs, they were stimulated to form spheroids, which are used as a marker of stem cell functionality. Figure 6 illustrates the formation of 3D spheroids derived from MSCs over two different time points: day 3 and day 5. In the positive control (shown in the left column), the spheroids were dense and compact, indicating strong cell–cell aggregation and healthy cell interactions. These spheroids maintained a consistent shape, suggesting robust cellular cohesion. The resulting spheroids after treatment with PFL for 5 min (shown in the right column) also exhibited compact structures, although they appeared slightly more irregular in shape compared to the control. Despite these minor irregularities, the spheroids maintained a generally cohesive structure and size similar to the spheroids grown in the presence of antibiotics. This indicates that isolated MSCs of PFL-rinsed pUCs maintain their functional capacity as stem cells and that these cells are suitable for complex tissue engineering.

## 4. Discussion

In this study, we successfully developed a process of rinsing with PFL as an alternative to antibiotics for disinfecting pUCs and subsequently isolating viable MSCs. The workflow developed within this study is presented in a graphical summary. This depicts the combination of PFL rinsing and ultrasound application from the initial rinsing of the pUC to the formation of spheroids from the isolated, functional MSCs. This investigation thereby provides the prerequisite for using pUCs as a sustainable and ethically accepted stem cell source for cell-based meat production without the use of antibiotics.

MSCs derived from pUCs offer several advantages over those from other sources. As well as being collected non-invasively, the abundance of pUCs as a by-product of birth ensures a scalable and renewable source of MSCs, making them suitable for large-scale production. Furthermore, UC-derived MSCs demonstrate superior proliferation and multilineage differentiation potential, allowing the generation of muscle, fat, and connective tissues that are crucial for replicating meat structures [3,33,34,35]. The challenge is to find an effective decontamination process that aligns with the goal of healthy and sustainable food production. Along this line, PFLs were investigated as an alternative decontamination strategy to replace antibiotics in the process. Building upon our previous findings [15], we implemented an extended approach that used a microwave-driven plasma source for PFL generation and combined it with ultrasound treatment. Among the described strategies, PFL rinsing with only mechanical shaking achieved sterility through effective bacterial removal facilitated by vigorous mechanical agitation and the chemical action of PFL. PFL, enriched with long-lived reactive species (H_2_O_2_, NO_3_^−^, and NO_2_^−^) at low pH (1.99), contributed to microbial inactivation [36,37,38], while the intense shaking dislodged bacteria. However, it is important to note that the aforementioned components also introduced multiple stress factors, including but not limited to excessive mechanical stress, chemical stress through reactive oxygen and nitrogen species, and low pH. PFL exhibited a strong acidic pH of 1.99, in contrast to the 5.66 recorded in the NaCl solution (negative control). These stressors compromised pUC tissue integrity and reduced the cell viability and yield of isolated MSCs. Furthermore, the presence of reactive species, such as NO_3_^−^ and NO_2_^−^, is regulated in food products, and further analysis is required to evaluate residual reactive species and address potential food safety concerns when utilizing these cells in the production of cultured meat products.

Conversely, ultrasound-assisted rinsing utilizes cavitation and microstreaming effects to gently dislodge bacteria from tissue surfaces. Enhanced antimicrobial action has been attributed to enhanced transport and penetration of reactive species [27,28,39]. The collapse of cavitation bubbles created localized shear forces that aided bacterial detachment without subjecting the pUCs to excessive mechanical stress. However, ultrasound-assisted rinsing alone was inadequate for complete sterilization, as its milder shear forces did not compensate for the lower bacterial detachment efficiency compared to shaker-assisted rinsing.

Further, the superior performance of the herein tested microwave-driven plasma device can be explained by its mode of action. Unlike direct-contact plasma–liquid systems, the microwave-driven plasma source generates plasma-processed air (PPA) that is bubbled into liquids post-discharge, allowing precise control of dissolved reactive species via gas–liquid mass transfer. The generated PFL showed higher H_2_O_2_ concentrations compared to previously tested plasma devices [15,40] and a rapid acidification (pH 1.99 within 4 min vs. pH 2.63 within 60 min in a spark discharge (wINPlas XXL)), which strongly contributed to the enhanced antimicrobial efficacy. Spark discharge systems, by contrast, produced negligible H_2_O_2_ and much lower NO_3_^−^ levels. Additionally, the two-stage microwave excitation allows fine control of effluent composition (NO_x_, O_3_) and enables scalable PFL production (4 L in 4 min) with excellent chemical stability and batch reproducibility.

In order to optimize the balance between pUC sterility and MSC viability, the utilization of PFL in conjunction with alternating ultrasound- and shaker-assisted treatment has been demonstrated to be highly effective, achieving superior microbial load reductions of 4.08 to 7.41 log_10_ CFU/pUC without compromising MSC viability. It was demonstrated that decreased duration of rinsing cycles also resulted in enhanced cell recovery. A five-minute rinsing cycle resulted in a 1.20-fold increase in cell viability and a 4.78-fold increase in cell yield when compared to a 10 min cycle, demonstrating the importance of optimizing rinsing duration to balance sterility and MSC recovery. By integrating mechanical agitation, cavitation-induced shear forces, and chemical decontamination mechanisms, the alternating ultrasound- and shaker-assisted PFL rinsing strategy resulted in complete decontamination and a 21.1% increase in MSC yield (5.45 × 10^5^ cells/g ± 1.94) compared to just ultrasound-assisted rinsing and 3.19-fold higher number of viable cells compared to a protocol without additional measures [15]. Our approach achieved cell yields of 5.45 ± 1.94 × 10^5^ cells/g, outperforming Zhu et al. [41], who reported 5.3 × 10^4^ muscle stem cells per gram of neonatal pig muscle tissue.

In the context of ultrasonic cleaning applications, the choice of frequency, power density, and time is of paramount importance for ensuring effective decontamination while concomitantly minimizing potential cellular damage. As demonstrated by Zhao et al., the decontamination of fish was enhanced following US treatment for 10 min at a low frequency (25 kHz). This treatment did not result in any alterations in the quality of the fish [42]. For decontaminating fruits and vegetables by ultrasound, a 10-min treatment was frequently reported, as reviewed by Bilek et al. [43], suggesting that this duration was feasible. Further, de Lucas et al. [44] reported that optimal low-intensity pulsed ultrasound (LIPU) application did not alter the proliferation, morphology, or cytoskeletal organization of mesenchymal precursors, nor did it affect their migration and invasion capabilities. Additionally, LIPU has been shown to potentially enhance the chondrogenic differentiation of MSCs [45]. In this study, we employed an ultrasonic bath operating at 35 kHz and a nominal power of 160 W, which is commonly used for ultrasonic extraction and cell lysis due to its strong mechanical effects. While LIPU does not damage mesenchymal precursors [44], the frequency and ultrasonic power applied in this study might have mechanical effects on the tissue. However, the protective barrier of the pUCs (Wharton’s jelly and outer membrane) serves as a buffer, reducing direct mechanical and chemical impact on embedded MSCs [46]. Additionally, transferring PFL-rinsed pUCs to a nutrient-rich cell culture medium (supplemented with 10% FCS) prior to explant culture preparation was crucial for cell recovery and primary stem cell isolation. The recovery-enhancing effects of FCS in culture medium can be attributed to its rich composition of antioxidants (glutathione) and key enzymes, such as superoxide dismutase (SOD) and catalase [47,48], which have been shown to reduce PFL-mediated oxidative stress. In order to further minimize the pH stress on explant tissue, a final wash in 10-fold diluted PFL (pH 3.05) was applied. It is evident from the findings of this study that the optimization of the duration of ultrasound- and shaker-assisted PFL rinsing, followed by immediate transfer to recovery media, is an effective method for preserving the viability of MSCs while achieving robust decontamination.

Our study addressed naturally contaminated samples as opposed to using artificially inoculated samples, such as those examining the synergistic effects of ultrasound and plasma-activated water (PAW) for microbial inactivation in chicken meat and skin [29]. Natural contamination presents greater microbial species variability, diverse colonization patterns, and unknown bacterial variations among test specimens. While artificial inoculation simplifies microbial interactions and distribution, natural contamination introduces real-world complexity, making effective decontamination more challenging. However, it also ensures that the developed PFL decontamination strategies are relevant for future industrial applications without overestimation. The high-throughput plasma device used in this investigation has previously been successfully employed in the industrial-scale washing process of fresh produce, such as lettuce, achieving significant microbial reduction without compromising product quality [30,31]. Our study extends these findings on utilizing microwave-driven plasma-generated PFL to more complex biological tissue derived from animals, albeit with five times longer rinsing times. This highlights the broader relevance of PFL-based processing in cellular agriculture.

## 5. Conclusions

This study established the feasibility of PFL generated by a microwave-driven plasma device in combination with ultrasound- and shaker-assisted rinsing to enable antibiotic-free decontamination of pUC tissue for viable MSC isolation. The final refined protocol achieved complete microbial elimination under the investigated conditions while maintaining high MSC viability and functional capacity, as validated by successful isolation of primary cells (2D) and subsequent spheroid formation (3D). These findings support the integration of PFL rinsing as an alternative to antibiotics for downstream applications, such as in cultured meat bioprocesses.

While PFLs inherently contain NO_3_^−^ and NO_2_^−^, which are strictly regulated in food products due to potential health risks, the present work focused on early-stage tissue decontamination and stem cell isolation. Although this study did not evaluate residual reactive species, the upstream nature of the process requires further analysis to address potential food safety concerns.

This study highlights the scalability of PFL-based methods, providing a sustainable, antibiotic-free alternative to conventional sterilization techniques. As regulatory frameworks tighten for the utilization of antibiotics in cellular agriculture, PFL could serve as a viable industry-ready solution for ensuring microbial safety while supporting ethically sourced, high-quality stem cells for cultured meat production.

## Figures and Tables

**Table 1:** Concentration of reactive species across three batches of generated PFL.

Parameter	Concentration
Hydrogen peroxide (H_2_O_2_)	0.165 (±0.01) mM
Nitrite (NO_2_^−^)	6.46 (±0.04) mM
Nitrate (NO_3_^−^)	7.03 (±0.05) mM
pH value	2.05 (±0.03)

**Table 2:** Overview of all experimental conditions iteratively applied in this study. Decontamination scenarios included (1) PFL rinsing assisted by shaking, (2) PFL rinsing assisted by shaking and a subsequent incubation in recovery solutions to improve the cell viability, (3) PFL rinsing assisted by ultrasound, and (4) PFL rinsing assisted by alternating combination with ultrasound and shaking. The table summarizes all variations in solutions for transportation, pre-rinsing steps, recovery solutions, and post-rinsing steps. The respective variables to optimize treatment parameters are highlighted in bold. Abbreviations: PFL: plasma functionalized liquid, pUC: porcine umbilical cord, FCS: fetal calf serum, α-MEM: minimum essential medium.

Transport	Trial Defined Mode	Cut Ends of pUC	Pre-Rinsing					Recover Solution	Cut Ends of pUC	Post-Rinsing During Creating Explant Culture		
			Solution	Volume	Assisted by	Time	Rounds			Solution	Volume	Rounds
NaCl solution	**Negative control**	No	NaCl	100 mL	shaker	10 min	3×	-	-	-	-	-
	**PFL** from microwave-driven plasma source (4 L NaCl solution/4 min)	***(1) shaker-assisted PFL rinsing:***										
		No	PFL	100 mL	shaker	**1 min**	3×	PFL	Yes	PFL	20 mL	4×
		No				**5 min**	3×	PFL	Yes	PFL	20 mL	4×
		No				**10 min**	3×	PFL	Yes	PFL	20 mL	4×
		***(2) shaker-assisted PFL rinsing with recover solution:***										
		No	PFL	100 mL	shaker	10 min	3×	**0.9% NaCl (4h)**	Yes	10-fold dilution of stock PFL	20 mL	1×
		No				10 min	3×	**α-MEM with FCS (4 h)**	Yes	10-fold dilution of stock PFL	20 mL	1×
		No				10 min	3×	**α-MEM with FCS (24 h)**	Yes	10-fold dilution of stock PFL	20 mL	1×
		***(3) ultrasound-assisted PFL rinsing:***										
		No	PFL	100 mL	**ultrasonic bath (50%)**	**5 min**	3×	α-MEM with FCS	Yes	10-fold dilution of stock PFL	30 mL	1×
		No				**10 min**	3×	α-MEM with FCS	Yes	10-fold dilution of stock PFL	30 mL	1×
		No			**ultrasonic bath (100%)**	**5 min**	3×	α-MEM with FCS	Yes	10-fold dilution of stock PFL	30 mL	1×
		No				**10 min**	3×	α-MEM with FCS	Yes	10-fold dilution of stock PFL	30 mL	1×
		***(4) combined ultrasound- and shaker-assisted PFL rinsing:***										
		Yes	PFL	100 mL	**ultrasonic bath (100%)/shaker**	**5 min**	3×	α-MEM with FCS	No	10-fold dilution of stock PFL	30 mL	1×
		Yes				**10 min**	3×	α-MEM with FCS	No	10-fold dilution of stock PFL	30 mL	1×
NaCl solution + AB	**Positive control**	-	-	-	-	-	-	-	Yes	NaCl solution + AB	20 mL	4×

## Data Availability

The datasets presented in this article are not readily available because the curation process is still ongoing as of February 2026. Requests to access the datasets should be directed to [49].

## Abbreviations

The following abbreviations are used in this manuscript:

PFL	Plasma functionalized liquid
MSCs	Mesenchymal stem cells
US	Ultrasound
NaCl	Sodium chloride
FBS	Fetal bovine serum
IBC	International bulk container
pUC	Porcine umbilical cord
PPA	Plasma processed air
slm	Standard liter per minute
CI	Confidence interval
DPBS	Dulbecco’s phosphate-buffered solution
CFU	Colony forming unit
mL	Milliliter
LOD	Limit of detection
ROS	Reactive oxygen species
LIPU	Low-intensity pulsed ultrasound
